# Preoperative Ascorbic Acid Levels in Proximal Femur Fracture Patients Have No Postoperative Clinical Impact, While Ascorbic Acid Levels upon Discharge Have a Major Effect on Postoperative Outcome

**DOI:** 10.3390/jcm9010066

**Published:** 2019-12-26

**Authors:** Katharina Hill-Mündel, Johannes Schlegl, Hans Konrad Biesalski, Sabrina Ehnert, Steffen Schröter, Christian Bahrs, Donatus Nohr, Andreas K. Nüssler, Christoph Ihle

**Affiliations:** 1 Institute of Nutritional Science, University of Hohenheim, 70599 Stuttgart, Germany; Katharina_Hill@uni-hohenheim.de (K.H.-M.); biesal@uni-hohenheim.de (H.K.B.); donatus.nohr@uni-hohenheim.de (D.N.); 2 Siegfried Weller Research Institute, Department of Trauma and Reconstructive Surgery, Eberhard Karls University Tuebingen, BG Trauma Center Tuebingen, 72076 Tuebingen, Germany; johannes-georg.schlegl@student.uni-tuebingen.de (J.S.); sabrina.ehnert@med.uni-tuebingen.de (S.E.); sschroeter@bgu-tuebingen.de (S.S.); cbahrs@bgu-tuebingen.de (C.B.); andreas.nuessler@gmail.com (A.K.N.)

**Keywords:** proximal femoral fracture, ascorbic acid, oxidative stress, reactive oxygen species, malnutrition, NRS, MNA, geriatric patients

## Abstract

Proximal femur fractures show a high prevalence in elderly patients and are associated with an elevated risk of multimorbidity and early mortality. Recovery is impaired by malnutrition and oxidative stress, which is affected by antioxidants such as ascorbic acid (AA). This study monitored AA levels during hospitalization of patients with a proximal femur to investigate the influence of AA status to the clinical outcome. AA levels of 25 elderly patients with a proximal femur fracture were measured during hospitalization using high performance liquid chromatography. Plasma samples were collected preoperatively, on the first day after surgery, on the third day after surgery and on the day of discharge. Nutritional Risk Screening 2002 (NRS) and Mini Nutritional Assessment (MNA) were assessed to evaluate the nutritional status. In patients with proximal femoral fractures, preoperative AA concentrations were significantly lower compared to elderly patients without an acute fracture. A significant decrease of 33.8% in AA plasma level was measured on the day after surgery with a significant recovery up to the time of discharge. The preoperative AA status did not have any significant effect on clinical outcome. However, inadequate AA levels (<50 µmol/L) upon discharge significantly increased the incidence and the severity of postoperative complications. These results indicate that the AA status upon discharge has a greater impact on clinical outcome than assumed, and therefore, AA supplementation during hospitalization should be considered.

## 1. Introduction

Proximal femoral fractures (PFF) are one of the most common reasons for hospital admission among the elderly population and are associated with an increased risk of morbidity and early mortality [1,2,3]. At hospitalization, geriatric patients are often malnourished. The prevalence of hospital malnutrition is stated between 20–50% and even higher in elderly patients [4]. Malnutrition among elderly inpatients leads to prolonged hospital stays and higher complication rates, which increase costs for the health system [5,6,7].

Besides a suboptimal nutritional status, oxidative stress also negatively affects recovery after surgery [8]. Increased levels of oxidative stress occur acutely after orthopedic surgical procedures and trauma [8]. This is particularly relevant in geriatric patients, in which levels of reactive oxygen species (ROS) are elevated, and antioxidant defenses are diminished [8]. ROS production contributes especially to bone remodeling by promoting bone resorption. This leads to decreased bone mineral density (BMD), a common feature of osteoporosis, which in turn is a risk factor for PFF [9,10,11].

Increased oxidative stress is frequently associated with reduced ascorbic acid (AA) plasma concentrations [12,13]. AA acts as an antioxidant at physiological concentrations. It donates two electrons while undergoing two consecutive and reversible oxidations forming ascorbate free radical as an intermediate and dehydroascorbic acid. Being a very potent scavenger of ROS, AA prevents cellular damage induced by oxidative stress [14]. Moreover, AA is essential for collagen synthesis as a cofactor for hydroxylation of proline and lysine [15]. AA is also involved in bone formation and remodeling. It induces chondrogenic and osteogenic differentiation, proliferation of early stage osteoclasts and cell death in late stage osteoclasts [16]. Deficiency of AA adversely affects fracture healing in elderly AA deficient osteogenic disorder Shionogi rats [17]. Furthermore, AA deficiency is associated with an increased risk of hip fracture [18,19].

Previous studies mainly focused on the association between AA and fracture risk or nutritional status without measuring the exact AA status of the patients. However, Rodemeister et al. demonstrated an association between AA, dehydroascorbic acid and ascorbyl radicals in patients undergoing cardiovascular surgery, i.e., decreased AA plasma levels pre-, peri- and up to 7 postoperative days [12]. 

This study aimed to examine the relationship between the nutritional status in elderly orthopedic PFF patients using NRS 2002 and MNA and the pre- and postoperative plasma AA levels. In addition, the association of AA with clinical outcome and postoperative complications was investigated.

## 2. Experimental Section

### 2.1. Ethics Statement

The study, including patient material, was performed in accordance with the Declaration of Helsinki (1964) in its latest amendment. Patient survey and collection of the clinically relevant data was performed in accordance with the ethical vote under the project No. 429/2014BO2. All study participants have signed a written informed consent.

### 2.2. Patient Population and Surveys

The patient population was divided into three groups. Control group 1 (CG1) included young (<30 years), healthy non-smoking patients without chronic comorbidities and without a recent fracture. Control group 2 (CG2) consisted of elderly patients (>65 years) without a recent fracture. The age within CG 2 ranged between 67 and 87 years. Since CG 2 illustrates the elderly patients in average health, patients with diabetes mellitus, smokers and tumor patients were excluded. Elderly patients (>65 years) who suffered from a fracture of the proximal femur within the last 24 h were included in the study group (SG).

Upon admission all participants were given standardized questionnaires, concerning their nutritional status and general health. The Nutritional Risk Screening 2002 (NRS) and Mini Nutritional Assessment (MNA) were used to evaluate the nutritional status of the participants as they are widespread and established screening tools. In addition, the “food score Hohenheim” was used to asses eating habits more precisely. The “food score” quantifies the consumption of specific food items during an exact time frame. The results of the food score were divided into three nutritional categories to establish a nutritional status. Follow-up interviews were conducted on patients of the SG after 6–8 weeks and 12 months. These follow-up interviews included a general survey concerning postoperative complications and a nutritional survey including the MNA, NRS and the food score Hohenheim.

The postoperative complications were graded based on the Clavien-Dindo classification as shown in Table 1 [20,21].

### 2.3. Blood Sampling and Ascorbic Acid Analysis

Plasma samples were collected at four different time points: preoperatively (T1), first day after surgery (T2), third day after surgery (T3) and on the day of discharge from hospital (T4). Shortly after blood sampling, ethylene diamine tetraacetic acid-Monovets (Sarstedt, Nümbrecht, Germany) were centrifuged at 1000× *g* for 5 min at 4 °C. To stabilize ascorbic acid, resulting plasma was aliquoted and diluted 1:1 with cold 5% perchloric acid (Carl Roth, Karlsruhe, Germany). Aliquots were instantly snap frozen in liquid nitrogen and stored at −80 °C. Blood sample preparation was always completed within a period of 20 min after sample collection.

For AA analysis, plasma samples were thawed at room temperature and centrifuged at 14,000× *g* for 5 min at 4 °C. As external standards, four different aqueous AA solutions (25 µM, 50 µM, 75 µM, 100 µM) were prepared and incubated with 5% perchloric acid. Samples and standards were diluted 2:1 with deionized water, centrifuged once again at 14,000× *g* for 5 min at 4 °C and transferred into brown vials (Th. Geyer GmbH & Co. KG, Renningen, Germany) for HPLC analysis. Sample and standard preparation was always performed on ice and light protected to avoid AA degradation. 

Analysis of samples was performed with HPLC using a reversed-phase column (Reprosil-Pur 120 C18 AQ 5 μM; Dr. Maisch HPLC GmbH, Ammerbuch, Germany) with 5 mM aqueous sodium phosphate buffer (pH 3.0) as mobile phase. The flow rate was 1.0 mL/min. Detection was performed by using a Coulochem II electrochemical detector (ESA, Chelmsford, United Kingdom) and a high-sensitivity analytical cell (Model 5011, ESA) at −300 mV (E1, upstream) and +300 mV (E2, downstream). Chromatograms were recorded and analyzed using software LabSolutions Version 5.54 (Shimadzu Deutschland GmbH, Duisburg, Germany). Table 2 shows AA value limits which were utilized to classify AA status [22]. 

### 2.4. Statistical Analysis

The data were analyzed by IBM SPSS Statistics 24 (SPSS Inc., IBM Company, Chicago, IL, USA). Depending on the scale level and the distribution of the data the following statistical tests were used: Kolmogorov-Smirnov-test, Shapiro-Wilk-test, Chi-Quadrat-test, McNemar-test, Mann-Whitney-U-test, unpaired t-test, Wilcoxon-sign-rank-test, paired t-test, Kruskal-Wallis-test, ANOVA, MANOVA and Friedman-test. For the rank correlation the significant value was set at *p* < 0.05. All graphs were created with GraphPad Prism 8 (GraphPad Software Inc., San Diego, CA, USA).

## 3. Results

### 3.1. Basic Clinical Parameters of the Study Population 

Twenty-five patients with proximal femoral fracture were included in the study group. CG 2 was reduced to 24 and CG1 to 15 patients due to statistical outliners. In the first 12 months after surgery 92% (23 patients) of the SG provided full information, whereas 8% (two patients) denied any further questioning. The mortality within SG was 34.8% over the course of the first postoperative year. Five out of 23 patients (21.7%) passed within 6–8 weeks after surgery, while 3/23 died during the rest of the year. Therefore, after 6–8 weeks, full information was received on 18 patients, and after 12 months 15 patients gave full information. The personal living conditions of SG after 6–8 weeks deteriorated significantly (*p* = 0.039) compared to the initial conditions. Although the living conditions after 12 months improved towards the first postoperative interview without a statistically significant relevance (*p* = 0.108). After 1 year there was no significant impairment (*p* = 0.108) in comparison to the preoperative living conditions.

The daily access to food was significantly limited (*p* = 0.007) in CG 2 in comparison to CG 1. There was no significant difference between CG 2 and SG concerning the food supply. After 6–8 postoperative weeks the food supply conditions have worsened significantly (*p* = 0.015). Although the food supply within the SG moderately improved after 12 months, statistical significance was not given (*p* = 0.059). There was no significant change (*p* = 0.105) in the access of daily food between the 12 months interview and the initial preoperative situation.

The mobility within CG 1 was significantly better (*p* = 0.038) than within CG 2 and within the SG (*p* < 0.001). At T4 the mobility within SG was significantly limited (*p* < 0.001) due to prior surgery. After 6–8 weeks the patients were significantly more immobile (*p* < 0.001) in comparison to T1. After 12 months the mobility within the SG has significantly improved in comparison to T4 (*p* = 0.008) and to the 6–8-week interview (*p* = 0.018). Patients did not regain preoperative mobility after 12 months (*p* = 0.006).

The level of care did not change significantly 6–8 weeks after surgery (*p* = 0.059). Although the level of care did improve in comparison to the first postoperative interview, this improvement was not significant (*p* = 0.334). The preoperative level of care was significantly less extensive than after 12 months (*p* = 0.008). Table 3 is an overview on the study population.

### 3.2. Ascorbic Acid Plasma Concentration

**Figure 1:**
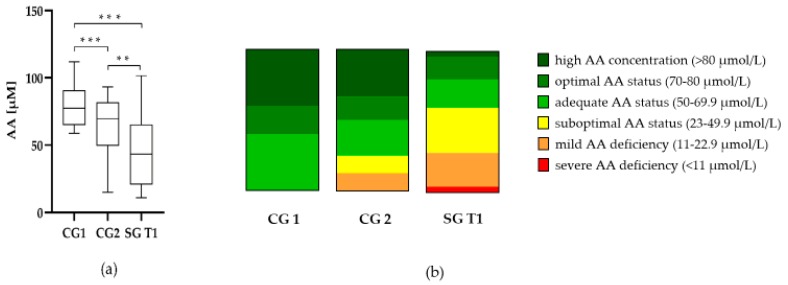
(**a**) AA (ascorbic acid) plasma concentrations and (**b**) AA status of CG1, CG2 and SG at T1 (preoperative). ** *p* < 0.01 and *** *p* < 0.001. CG: control group SG: study group.

The AA plasma concentration in CG 1 (79.56 ± 16.70 µmol/L) was significantly higher (*p* < 0.001) than both, CG 2 (62.76 ± 22.61 µmol/L) and SG at T1 (44.96 ± 24.05 µmol/L) (Figure 1). The AA plasma concentration in CG 2 was also significantly higher than in SG (*p* < 0.010). Patients within CG 1 showed an optimal AA status, while the average AA plasma concentration in CG 2 was adequate (Figure 1b). The average preoperative AA status within SG was classified as suboptimal. 

### 3.3. Ascorbic Acid Concentrations during Hospitalization 

**Figure 2:**
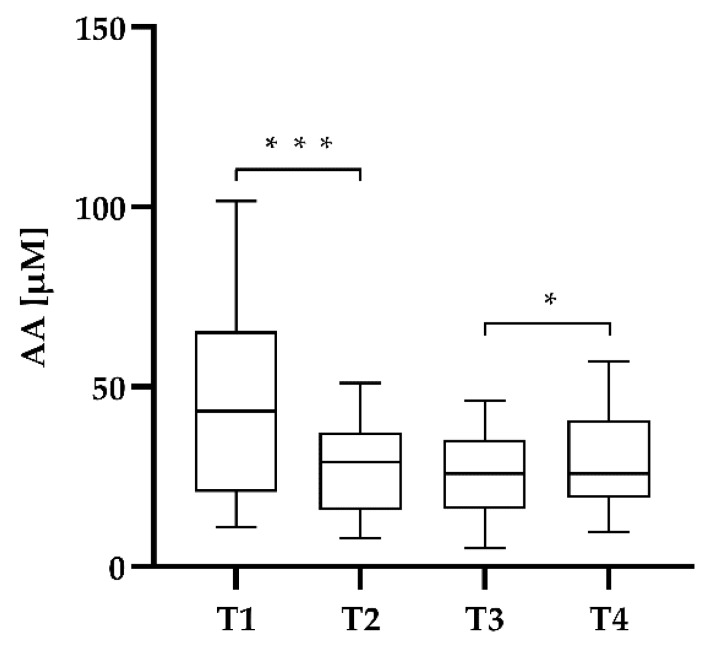
AA plasma concentrations of study group during hospitalization. Plasma samples were collected at four different time points: preoperatively (T1), first day after surgery (T2), third day after surgery (T3) and on the day of discharge from hospital (T4). * *p* < 0.05 and *** *p* < 0.001.

In SG, preoperative AA levels were 44.96 ± 24.05 µmol/L at T1 (Figure 2). On the first day after surgery a highly significant (*p* < 0.001) decrease by 33.68% to 27.61 ± 12.55 µmol/L at T2 (Figure 2) was observed. AA plasma levels did not decrease further between T2 and T3. On the day of discharge from hospital (T4) AA concentrations significantly (*p* = 0.013) increased (29.36 ± 13.16 µmol/L).

### 3.4. Delta 

This AA loss of between T1 and T2 was established as Delta and divided into three severity categories with statistically even groups. Limit values were set at −30.67% and −40.20% AA concentration at T2. The average AA reduction was 33.68%. Category 1 (<−30.67%) was established as mild AA loss. Values between −30.67% and −40.20% were labelled as moderate AA loss. Category 3 (>−40.20) was established as severe AA loss. The majority of SG (36%) suffered a moderate loss. Category 1 and 3 each contained 32% of SG. Hence, 66% of SG lost >1/3 of their initial AA plasma concentration during surgery. The AA value of 32% of patients even dropped by >2/5 of their initial value.

### 3.5. NRS Status

**Figure 3:**
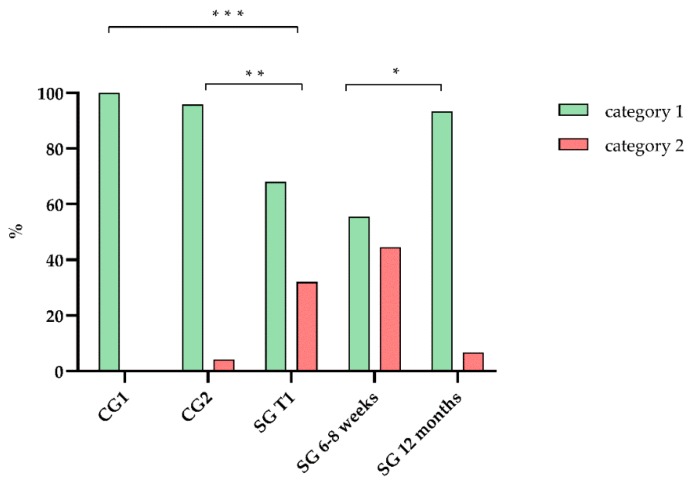
NRS (Nutritional Risk Screening 2002) categories of CG 1, CG 2 and SG at different time points. Category 1: well-nourished (NRS score < 3) and category 2: nutritionally at risk (NRS score ≥ 3). * *p* < 0.05, ** *p* < 0.01 and *** *p* < 0.001.

Although the participants of the CG 1 scored significantly higher (*p* < 0.001) than those in the CG 2, there was no significant difference (*p* = 0.423) in the NRS status between them (Figure 3). The preoperative NRS status of SG was significantly worse than in CG 2 (*p* = 0.023) and CG 1 (*p* = 0.016). The postoperative NRS status after 6–8 weeks was not significantly worse (*p* = 0.480) than prior to surgery. In comparison to the first postoperative interview a significant improvement (*p* = 0.025) of the NRS status was observed after 12 months. Furthermore, there was no significant change (*p* = 0.180) in NRS status in comparison to T1.

### 3.6. MNA Status

**Figure 4:**
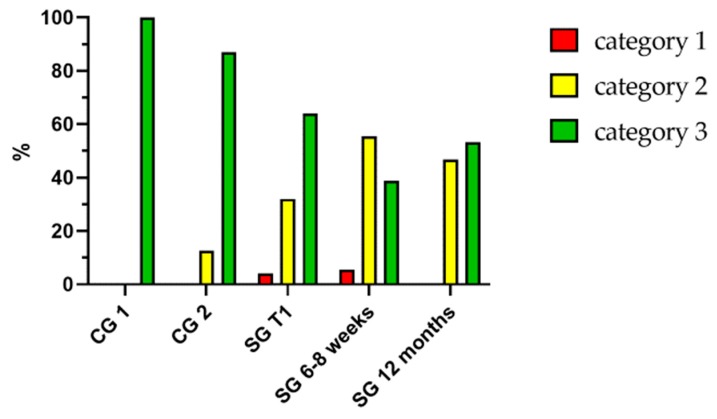
MNA (Mini Nutritional Assessment) categories of CG 1, CG 2 and SG at set time points. Category 1: malnourished (<17 pts.), category 2: risk of malnutrition (17–23.5 pts.) and category 3: well-nourished (24–30 pts.).

There was no significant difference (*p* = 0.154) regarding the MNA status between CG 1 and CG 2 (Figure 4). However, the MNA status in CG 1 was significantly better (*p* = 0.009) compared to the MNA status within SG. The MNA status did not worsen significantly (*p* = 0.206) after 6–8 weeks nor after 12 months in comparison to the first postoperative interview (*p* = 0.180) or T1 (*p* = 0.317).

### 3.7. Food Score

The elderly patients within CG 2 (62.0 ± 7.8/100 pts.) scored significantly higher (*p* = 0.037) than the young participants within the CG 1 (54.9 ± 13.0/100 pts.). However, there was no significant difference (*p* = 0.168) between the two groups regarding the nutritional status. Patients within SG (54.7 ± 11.1/100 pts.) did not score significantly lower (*p* = 0.970) than CG 1. The nutritional status did not reveal any significant differences (*p* = 0.263) between SG and CG 2, although SG patients scored significantly lower (*p* = 0.011) than participants in CG 2. The nutritional status of the SG and the CG 1 did not show any significant discrepancies either (*p* = 0.664). 

### 3.8. Postoperative Complications

The postoperative complications were pooled into three severity levels, shown in Table 4, using the Clavien-Dindo classification, due to the low headcount within SG to facilitate statistical analysis [20,21]. 

Specific orthopedic complications occurred rarely during the postoperative period. A cut out of the locking screw was reported by one patient (4%), who therefore needed re-operation. One patient (4%) suffered from prothesis infection and wound healing deficit also resulting in re-operation. No nonunions were reported. Complications in general were more common during the stationary phase of the rehabilitation. These stationary complications included common infections such as pneumonia within three patients (12%) and uriniary tract infection within four patients (16%). Additionally, two cases (8%) of postoperative thrombosis with consecutive pulmonary embolism were reported. Perioperative anemia due to excessive blood loss occurred in most of the cases (88%), yet a perioperative blood transfusion was only required on two occasions (12%). Severe postoperative anemia with the necessity of a transfusion was recorded in seven (28%) cases.

**Figure 5:**
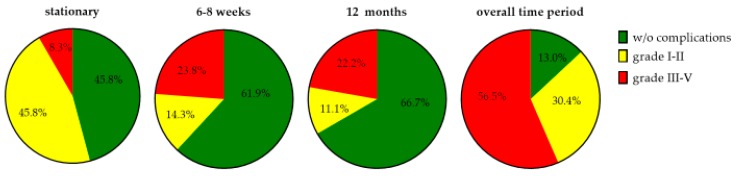
Postoperative complications of SG at different time points according to the Clavien-Dindo classification [20,21].

Neither the occurrence (Figure 5) nor the severity of a postoperative complication displays any significant differences regarding the time of the interviews. No significantly higher occurrence (*p* = 0.388) or increased severity (*p* = 0.922) could be detected between the stationary time period and the 6–8 week follow up interview. No significant difference regarding the occurrence (*p* = 1.000) or the severity (*p* = 0.435) could be observed between the first and the second postoperative interview. 

#### 3.8.1. Postoperative Complications and AA Status 

There was a weak (*r* = −0.224) but insignificant (*p* = 0.305) correlation between the preoperative AA status and the severity of postoperative complications within 12 months. The occurrence of such complications showed insignificant coherence (*p* = 0.096) with the AA status at T1. 

A significant connection (*p* = 0.016) between the postoperative AA status at T2 and the occurrence of stationary postoperative complications could be demonstrated. The severity of such a stationary complication and the postoperative AA status at T2 correlated moderately (*r* = −0.471) and significantly (*p* = 0.020). 

There was a significant connection (*p* = 0.012) between the occurrence of stationary postoperative complications and the AA status at T3. The severity of a stationary postoperative complication also correlated strongly (*r* = −0.547) and significantly (*p* = 0.006) with the AA status on the third postoperative day. AA deficiency at T3 also correlated strongly (*r* = 0.559) and significantly (*p* = 0.005) with the occurrence of a stationary postoperative complication.

There was a moderate (*r* = 0.438) and significant (*p* = 0.047) coherence between a deficient AA status at T4 and the occurrence of a postoperative stationary complication. The AA status upon discharge correlated strongly (*r* = −0.514) and significantly (*p* = 0.014) with the severity of a postoperative complication during the entire 12-month period.

#### 3.8.2. Postoperative Complications and NRS Status

There was a significant connection (*p* = 0.007) between the NRS status after 6–8 weeks and the occurrence of a postoperative complication after 6–8 weeks. The severity of these complications showed a strong (*r* = 0.685) and significant (*p* = 0.002) correlation to the NRS status after 6–8 weeks. The NRS status after 6–8 weeks also showed a significant (*p* = 0.043) coherence to the occurrence of complications after 12 months. There was also a strong (*r* = 0.534) and significant (*p* = 0.022) correlation between the NRS status after 6–8 weeks and the severity of a postoperative complication after 12 months. The NRS status after 12 months correlates strongly (*r* = 0.576) and significantly (*p* = 0.025) with the severity of a postoperative complication after 12 months. 

#### 3.8.3. Postoperative Complications and MNA Status

There was a significant (*p* = 0.049) connection between the MNA status after 6–8 weeks and the occurrence of a postoperative complication in the first 6–8 weeks. The severity of that postoperative complication correlated strongly (*r* = −0.575) and significantly (*p* = 0.012) with the MNA status after 6–8 weeks.

#### 3.8.4. Postoperative Complications and Clinical Parameters

The preoperative access to food correlated strongly (*r* = 0.519) and significantly (*p* = 0.011) with the magnitude of a postoperative complication. There was also a strong (*r* = 0.529) and significant (*p* = 0.042) coherence between the daily access to nutrition after 12 months and the severity of a postoperative complication. The preoperative level of care displayed a strong (*r* = 0.537) and significant (*p* = 0.008) connection towards the severity of a postoperative complication. 

The postoperative mobility upon discharge correlated strongly (*r* = 0.522) and significantly (*p* = 0.013) with the severity of a postoperative complication. The mobility at T4 also correlated moderately (*r* = 0.439) and significantly (*p* = 0.047) with the magnitude of a postoperative complication after 6–8 weeks. A significant (*p* = 0.025) connection between the mobility after 6–8 weeks and the occurrence of a postoperative complication could be detected. Additionally, the dimension of such complications displayed a strong (*r* = 0.562) and significant (*p* = 0.015) coherence towards the mobility after 6–8 weeks.

#### 3.8.5. Mortality within 12 Months after Surgery

Mortality could not be significantly linked to neither preoperative (*p* = 0.314) nor postoperative AA status at T2 (*p* = 0.560). There was also no significant connection to the AA status upon discharge (*p* = 0.802). Mortality also did not correlate with Delta (*p* = 0.848), the preoperative NRS status (*p* = 0.371) or the preoperative MNA status (*p* = 0.343). However, there was a significant connection (*p* = 0.009) between the mobility upon discharge and the mortality within 6–8 weeks and within 12 months (*p* = 0.005). The mortality within 6–8 postoperative weeks showed significant connections to the preoperative living circumstances (*p* = 0.046) as well as the preoperative access to food (*p* = 0.031) and the preoperative level of care (*p* = 0.017). 

### 3.9. Clinical Parameters

Prior to surgery the nutritional status correlated strongly (*r* = 0.563) and significantly (*p* = 0.003) with the level of care. There was a moderate (*r* = 0.448) and significant (*p* = 0.025) coherence between the access of food and the preoperative nutritional status based on the food score. The access of daily nutrition correlated moderately (*r* = −0.411) and significantly (*p* = 0.041) with the AA status at T1. There was a strong (*r* = 0.568) and significant (*p* = 0.003) coherence between the preoperative AA status and Delta.

While the NRS status failed to reveal any significant connections to the AA status, there was a strong (*r* = 0.511) and significant (*p* = 0.011) correlation between the preoperative MNA status and the AA status at T4. The AA status upon discharge also correlated strongly (*r* = 0.653) and significantly (*p* = 0.003) with the MNA status after 6–8 weeks. AA deficiency at T4 displayed a significant connection to both the MNA status after 6–8 weeks (*p* = 0.019) and the MNA status after 12 months (*p* = 0.041). 

There was a strong (*r* = −0.544) and significant (*p* = 0.017) coherence between the mobility of the patients upon discharge and the MNA status after 6–8 weeks. The MNA status after 6–8 weeks correlated even stronger (*r* = −0.761) and significantly (*p* < 0.001) with the mobility after 6–8 weeks. There was a strong (*r* = −0.527) and significant (*p* = 0.010) connection between the AA status at T4 and the mobility after 6–8 weeks. This correlation was still strong (*r* = −0.525) and significant (*p* = 0.025) after 12 months. The preoperative level of care strongly (*r* = 0.608) and significantly (*p* = 0.001) affected the preoperative mobility as well as the mobility upon discharge (*r* = 0.513) (*p* = 0.010).

After 6–8 postoperative weeks the access of daily nutrition correlated strongly (r = 0.652) and significantly (*p* = 0.003) with the living circumstances. The preoperative access to daily food was strongly (*r* = 0.692) and significantly (*p* < 0.001) influenced by the preoperative level of care. A moderate (*r* = −0.491) and significant (*p* = 0.039) coherence could be detected between the AA status upon discharge and the level of care after 6–8 weeks. The AA status upon discharge also correlated strongly (*r* = −0.570) and significantly (*p* = 0.026) with the level of care after 12 months. The preoperative level of care correlated strongly (*r* = −0.532) and significantly (*p* = 0.006) with Delta.

## 4. Discussion

This study monitored AA levels in elderly PFF patients during hospitalization. Preoperative AA plasma concentrations were compared to elderly patients without trauma and to postoperative AA levels to explore the effects of PFF and major surgery on AA plasma concentrations. The results were compared to previous papers including a similar study from Rodemeister et al., which monitored AA levels in patients following cardiac surgery [12]. AA concentrations were correlated to important clinical pre- and postoperative parameters to evaluate the impact on clinical outcome and postoperative complications.

### 4.1. Ascorbic Acid Status 

CG 1 averaged optimal AA concentrations with all participants reaching at least an adequate AA status. Therefore, CG 1 fulfills its purpose as the most basic reference in AA levels [22]. On average the older patients within CG 2 reached adequate AA levels. Although absolute AA concentrations in CG 1 were significantly higher than in CG 2, no significant difference in AA status could be detected. The likely reason for the lack of difference regarding the AA status is the small number of participants within CG 1. The significantly lower AA levels are most likely due to the higher age within CG 2. It is well known that among other micronutrients, AA plasma concentrations decline with higher age [23]. Decreased liver and kidney function [24] and the increasing number of chronic diseases [25], such as diabetes mellitus and hypertension [26], are examples for the negative impact of older age on AA saturation. The AA levels of CG 2 serve as a reference to preoperative SG levels since they represent the same age category, and therefore, have a similar lifestyle and preconditions. 

On average, preoperative AA levels within SG showed suboptimal AA concentrations. Furthermore, 24% of the SG had a mild AA deficiency and 4% of SG were severely deficient. Preoperative AA levels within SG were significantly lower than within CG 2 by >28% There are many reasons for these preoperative results. One of which is that the preoperative living conditions within the SG are less independent. Femur fracture patients more often live in a nursing home or in ambulatory care, which significantly limits their access to food and increases the risk of malnutrition [27] and AA deficiency [28]. Femur fracture patients within SG are significantly less self-sufficient than within CG 2. Furthermore, the level of care is significantly higher within SG, which is considered to be one of the highest risks for AA deficiency [27,29]. These connections can also be demonstrated in this study, since a moderate and significant correlation was found between the access of daily nutrition and the preoperative AA status within SG. In addition, both the preoperative level of care and the living conditions showed a moderate or respectively strong connection with access to nutrition [30,31]. Therefore, these two clinical parameters influence preoperative AA levels indirectly [32,33]. 

Patients within the SG suffered a fracture of the proximal femur within the last 24 h, which had the biggest impact on AA levels [34]. Reduced antioxidative parameters indicate an increased level of oxidative stress, which was triggered by the proximal femur fracture itself. A fracture of the femur and the bursting of collagen fibers contribute extensively to the immediate formation and rapid increase of ROS resulting in acute oxidative stress at the fracture site [8,35,36]. Oxidative stress is induced by lipid peroxides, free radicals and ROS, damaging a variety of cellular components including proteins, lipids within the cell wall and DNA. This could lead to increased cell death and vast tissue damage [8,37]. Geriatric patients within the SG are more affected, as age impairs the antioxidant power of the organism [28], and thus, causes higher levels of oxidative stress.

One of the most important results of this study is the massive AA loss during surgery. AA levels significantly dropped >33% between T1 and T2. Preoperative AA levels were significantly higher than postoperative AA levels at T2. Preoperatively, 40% of SG reached at least an adequate AA status, whereas at T2 only 4% displayed adequate AA status (> 50µmol/L). This severe decline in AA levels has been suggested to be due to the vast consumption of AA during the surgery, to counteract the increased oxidative stress. Severe drops in AA levels during surgery are comparable to the results found by Rodemeister et al. Patients in that study also showed a massive and significant AA loss of 41% during cardiac surgery [12]. Therefore, the surgery-associated results can be transferred to trauma patients. Since cardiac surgery as well as trauma surgery induces severe loss of AA, it can be assumed that major surgery triggers high levels of oxidative stress, rather than the usage of the cardiopulmonary bypass [8,38,39].

Rodemeister et al. monitored AA levels until discharge, finding no rebound or significant rise between the surgery and discharge after 7 days [12]. In contrast, in this study patients were discharged after 64 days, showing a significant rebound in AA levels upon discharge (T4). AA levels at T4 increased by >23% towards T3. This significant rebound upon discharge was not found in the paper published by Rodemeister et al., and therefore represents a distinct difference [12]. A possible explanation for this difference is that cardiac surgery strains the organism harder than an orthopedic surgery, resulting in a longer antioxidative recovery.

### 4.2. Clinical Outcome

Postoperatively, all patients with proximal femur fractures were interviewed twice (6–8 weeks and 12 months after surgery). In total, 87% of SG reported to have suffered a postoperative complication over the course of 12 months. Only 13% had no complications of any sort. The percentage of a postoperative complication seems excessive, since most of the studies report complication rates >30% [40,41], and only one paper showed similar results [42]. Since the surgical treatment of a proximal femur fracture has been improving over the last decades, some of the most feared orthopedic complications, such as nonunion, cutting out, prothesis infections or wound healing deficit, were rarely or never observed during the entire 12-month period. However due to the large percentages, postoperative complications were divided into three categories according to the Clavien-Dindo classification, to increase the clinical relevance on postoperative outcome. Category 0 complies to no complications, category 1 stands for mild complications (Clavien-Dindo classification 1,2) and category 3 complies to severe complications including death (Clavien-Dindo classification 3,4,5) [20,21]. In total, 56.52% suffered from a severe postoperative complication in the first postoperative year. These complications carry clinical relevance as they can prolong rehabilitation, impair the postoperative quality of live, lead to permanent disabilities and increase mortality [34,40,43]. The high rates of severe postoperative complications in this study can be explained with the low headcount of 25 fracture patients. The most important reason is, however, the old age within SG. These patients represent a very vulnerable population, being at enormous risk for postoperative complication and mortality during the first postoperative year [33,44]. In total, 40% of women and 25% of men died within 12 months after surgery. The overall mortality in this study was 34.78%. Due to the old age and subsequent multimorbidity the 1-year-mortality among even-aged elderly is 9.3% in women and 9.9% in men [45,46,47]. Many studies have shown similar results following proximal femur fracture [2,47,48,49], matching the mortality displayed in the study at hand.

### 4.3. Clinical Correlations to Postoperative Complications and Mortality

The severity of a postoperative complication within 1 year after surgery showed significant correlations towards preoperative clinical parameters such as access of nutrition and level of care. This concludes that an overall adequate self-sufficiency is advantageous to a successful rehabilitation [50]. Although preoperatively there were no significant connections between malnutrition and postoperative complication, 6–8 weeks after surgery there were strong correlations towards the MNA status and the NRS status. This shows a major influence of clinical parameters and malnutrition on the postoperative complications and rehabilitation [51]. Both scores are frequently used with geriatric patients. They have a high clinical validity towards malnutrition and daily routine [52], which makes these connections towards postoperative complications highly relevant for a successful rehabilitation [53,54].

**Figure 6:**
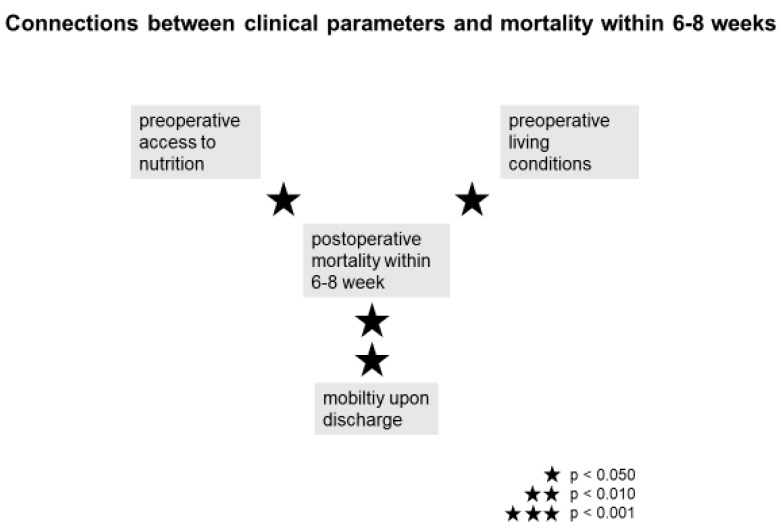
Correlation between clinical parameters and the postoperative mortality within 6–8 weeks.

Some of the clinical parameters influencing the postoperative mortality within 6–8 weeks following a proximal femur fracture are displayed in Figure 6.

Limited access to daily nutrition, impaired mobility and dependent living conditions prior to surgery facilitate postoperative mortality within 6–8 weeks after surgery. The mobility upon discharge has a major impact on postoperative survival. Preoperative improvements on these basic clinical parameters as well as supporting postoperative mobility and self-sufficiency significantly reduces postoperative mortality [45,51].

### 4.4. Clinical Findings Regarding the Preoperative AA Status

The preoperative AA status shows no correlation to the occurrence or the severity of a postoperative complication within the first year after surgery. AA levels at T1 do not show any significant influence on mortality, living conditions or mobility following surgery. These clinical parameters are crucial for a successful rehabilitation [45,55]. Therefore, the preoperative AA status does not show any significant impact on the postoperative clinical outcome in trauma patients in this study. The protective properties of an adequate preoperative AA status are presented by a few publications. Most of which demonstrate cardioprotective or pain-relieving effects in postoperative interviews [56,57,58]. Some papers also clearly line out the connection between malnutrition and AA deficiency and adverse clinical outcome, respectively [19,51,53]. These advantageous effects of adequate preoperative AA status on clinical outcome were not found in this study; therefore, extensive follow up studies with a much larger study group are required.

One of the most important findings of this study was the severe AA loss during surgery, which was defined as Delta. Delta displayed some relevant connections to AA status and other clinical parameters. The data showed a strong and highly significant connection between the AA status at T1 and Delta, which suggests that high AA plasma concentrations prior to surgery leads to a greater decline in AA levels. This hypothesis is confirmed by a significant connection between Delta and a mild and severe AA deficiency. Delta also showed strong preoperative correlations towards the access of nutrition and the level of care. Therefore, it seems that low levels of care and self-sufficiency regarding daily access of food result in high AA levels, which facilitates greater AA decline. A possible explanatory approach is, that a high antioxidative capacity is utilized by the organism to counteract ROS and therefore consumes large quantities of AA, whereas low antioxidative capacities are not being exhausted as much [57,59,60].

AA levels at T2 showed a significant connection to the occurrence and the severity of a stationary complication. Therefore, elevated AA levels on the first postoperative day seem to have protective effects during the stationary phase of rehabilitation. Nevertheless, no significant effects on the clinical outcome could be linked to an elevated AA status. AA levels at T2 also showed no significant correlation to postoperative mortality. However, there was a strong and significant correlation with the MNA status after 6–8 weeks, which suggests advantageous effects on the nutritional status and living circumstances. 

**Figure 7:**
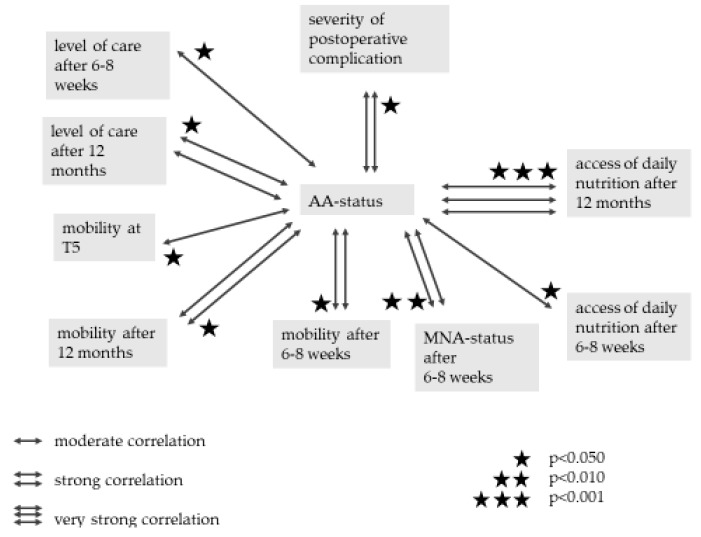
Impact of AA status at T4 (day of discharge from hospital) on clinical parameters.

Since one of the goals of this study was to measure and evaluate AA levels upon discharge regarding clinical outcome, postoperative focus shifted to T4. There are numerous clinical correlations to the AA status upon discharge, which are presented in Figure 7.

As mentioned before, there is a significant rebound in AA levels between T3 and T4. This re-increase in AA levels has beneficial postoperative effects on most of the clinical parameters. Thus, an elevated AA status not only has a strong beneficial effect on the severity of postoperative complications, but also affects the postoperative level of care and the access to daily nutrition. One of the most important factors of a satisfying rehabilitation is the postoperative mobility, which is also strongly benefited by an elevated AA status upon discharge.

Except for postoperative mortality, which shows no significant correlation, all relevant clinical parameters display protective or beneficial correlations to AA levels upon discharge. These clinical parameters are essential indicators of postoperative self-sufficiency and a successful rehabilitation [40,55]. The overall protective influence of elevated AA levels upon discharge is reinforced by its strong correlation to the MNA status after 6–8 weeks. This quantifiable clinical score for geriatric patients affirms beneficial effects on clinical outcome in fracture patients. These protective properties of an elevated AA status at T4 are not described in the literature, since most of the previous studies focused on pre- or perioperative AA levels [56] or neglected the impact of AA levels upon discharge on the clinical outcome [61].

Several results of this study show tremendous potential for clinical application, and therefore, need to be confirmed in order to raise clinical awareness and gain professional acceptance. Further replications of this study with a much larger study population are necessary to determine whether there are any postoperative benefits to an increased preoperative AA status. Further studies should also try to confirm the postoperative beneficial effects arising from AA levels at T2. The beneficial properties linked to an adequate AA status upon discharge seemed conclusive; nonetheless, they should remain the focus of future studies. Subsequently, placebo-controlled intervention studies should be conducted on a large population, to confirm protective effects on clinical outcome.

## 5. Conclusions

Although preoperative AA levels did not have significant effects on postoperative rehabilitation and complications in this study, the postoperative clinical findings regarding Delta and especially the AA status at T4 indicates clinical advantages of an AA supplementation. There are some publications outlining the protective and beneficial antioxidative effects of pre- and perioperative AA supplementation on postoperative clinical outcome in cardiac surgery [62], general surgery [63] and sepsis treatment [64,65]. The AA supplementation does not only have beneficial antioxidative effects, but also massive impact on bone metabolism [16,19,66,67]. Especially patients with fractures will most likely benefit from AA supplementation regarding bone growth, bone remodeling and fracture healing [17,68,69,70]. AA is easily produced in large quantities and therefore relatively cheap. It is generally accepted within the general population, which would result in a very high patient compliance. Furthermore, AA can be applied orally and intravenously.

AA should be supplemented orally as long-term medication in geriatric patients >65 years to maintain adequate AA levels >50 µmol/L. This constitutes as primary prophylaxis to strengthen bone metabolism and prevent major fractures. As secondary prophylaxis AA should be supplemented intravenously in high doses >3–6 g to restore normal plasma levels and counteract the oxidative stress induced by the fracture and the surgery [57,71]. After discharge, AA supplementation should be continued orally to ensure a successful rehabilitation.

## Figures and Tables

**Table 1:** Clavien-Dindo classification.

Clavien-Dindo Grade	Description
0	No complications.
1	Any deviation from the normal postoperative course without the need for pharmaceutical treatment other than the “allowed therapeutic regiments”, or surgical, endoscopic and radiological intervention.
2	Requiring pharmacological treatment with drugs beyond those allowed for grade I complications. Blood transfusions or total parenteral nutrition are also included.
3	Requiring surgical, endoscopic or radiological intervention.
4	Life threatening complication requiring critical care management.
5	Death.

**Table 2:** AA (ascorbic acid) classification.

Plasma Levels	AA Status
>80 µmol/L	high AA concentrations
70–80 µmol/L	optimal AA status
50–69.9 µmol/L	adequate AA status
23–49.9 µmol/L	suboptimal AA status
11–22.9 µmol/L	mild AA deficiency
<11 µmol/L	severe AA deficiency

**Table 3:** Overview on the study population. CG: control group SG: study group.

	CG 1	CG 2	SG T1	SG 6–8 Weeks	SG 12 Months
**Average Age in Years**	**22.93 ± 2.09**	**76.42 ± 6.52**	**83.96 ± 6.24**		
**Living conditions**					
Alone			8/25 (32.0%)	3/18 (16.7%)	3/15 (20.0%)
+1 person			12/25 (48.0%)	9/18 (50.0%)	10/15 (66.7%)
Nursing home			5/25 (20.0%)	3/18 (16.7%)	2/15 (13.3%)
Hospital			0/25 (0.0%)	3/18 (16.7%)	0/15 (0.0%)
**Access to food**					
Self-sufficient	100%	21/24 (87.5%)	12/25 (48.0%)	5/18 (27.8%)	7/15 (46.7%)
Private support	0%	2/24 (8.3%)	5/25 (20.0%)	3/18 (16.7%)	3/15 (20.0%)
Professional ambulatory aid	0%	1/24 (4.2%)	3/25 (12.0%)	3/18 (16.7%)	3/15 (20.0%)
Professional stationary support	0%	0/24 (0.0%)	5/25 (20.0%)	7/18 (38.9%)	2/15 (13.3%)
**Mobility**					
Walking	100%	18/24 (75.0%)	12/25 (48.0%)	0%	3/15 (20.0%)
Crutches	0%	2/24 (8.3%)	3/25 (12.0%)	5/18 (27.8%)	3/15 (20.0%)
Rollator	0%	3/24 (12.5%)	9/25 (36.0%)	7/18 (38.9%)	8/15 (53.3%)
Wheelchair	0%	1/24 (4.2%)	1/25 (4.0%)	3/18 (16.7%)	1/15 (6.7%)
Bedridden	0%	0/24 (0.0%)	0/25 (0.0%)	3/18 (16.7%)	0/15 (0.0%)
**Level of care**					
No care needed			16/25 (64.0%)	10/18 (55.6%)	7/15 (46.7%)
1st degree			5/25 (20.0%)	5/18 (27.8%)	3/15 (20.0%)
2nd degree			1/25 (4.0%)	0/18 (0.0%)	4/15 (26.7%)
3rd degree			3/25 (12.0%)	3/18 (16.7%)	1/15 (6.7%)

**Table 4:** Adaption of the Clavien-Dindo classification.

Severity Level	Description	Clavien-Dindo Grade
0	No complications	0
1	Mild complications	1, 2
2	Severe complications (including death)	3, 4, 5
